# Early Development of Resident Macrophages in the Mouse Cochlea Depends on Yolk Sac Hematopoiesis

**DOI:** 10.3389/fneur.2019.01115

**Published:** 2019-10-22

**Authors:** Ippei Kishimoto, Takayuki Okano, Koji Nishimura, Tsutomu Motohashi, Koichi Omori

**Affiliations:** ^1^Department of Otolaryngology, Head and Neck Surgery, Graduate School of Medicine, Kyoto University, Kyoto, Japan; ^2^Department of Tissue and Organ Development, Regeneration, and Advanced Medical Science, Graduate School of Medicine, Gifu University, Gifu, Japan

**Keywords:** resident macrophage, embryonic cochlea, Csf1r, Iba1, yolk sac, fetal liver, *in situ* proliferative capacity, immunohistochemistry

## Abstract

Resident macrophages reside in all tissues throughout the body and play a central role in both tissue homeostasis and inflammation. Although the inner ear was once believed to be “immune-privileged,” recent studies have shown that macrophages are distributed in the cochlea and may play important roles in the immune system thereof. Resident macrophages have heterogeneous origins among tissues and throughout developmental stages. However, the origins of embryonic cochlear macrophages remain unknown. Here, we show that the early development of resident macrophages in the mouse cochlea depends on yolk sac hematopoiesis. Accordingly, our results found that macrophages emerging around the developing otocyst at E10.5 exhibited dynamic changes in distribution and *in situ* proliferative capacity during embryonic and neonatal stages. Cochlear examination in *Csf1r*-null mice revealed a substantial decrease in the number of Iba1-positive macrophages in the spiral ganglion and spiral ligament, whereas they were still observed in the cochlear mesenchyme or on the intraluminal surface of the perilymphatic space. Our results demonstrated that two subtypes of resident macrophages are present in the embryonic cochlea, one being Csf1r-dependent macrophages that originate from the yolk sac and the other being Csf1r-independent macrophages that appear to be derived from the fetal liver via systemic circulation. We consider the present study to be a starting point for elucidating the roles of embryonic cochlear resident macrophages. Furthermore, resident macrophages in the embryonic cochlea could be a novel target for the treatment of various inner ear disorders.

## Introduction

Congenital hearing loss, which occurs in approximately one in one thousand newborns, is one of the many burdensome congenital anomalies or disabilities (1). In particular, cytomegalovirus (CMV) infection during the gestational period accounts for 15–21% of all congenital hearing loss cases (2). Although a large number of children develop hearing loss via congenital CMV infection every year, the detailed pathophysiology of CMV infection in the auditory pathway, including the cochlea, has not been fully understood. Moreover, no therapeutic treatment for congenital hearing loss due to prenatal viral infections, such as CMV or rubella virus, is currently present. To elucidate the pathophysiological mechanisms and develop effective methods for treating cochlear damage due to intrauterine infection, understanding the immune system of the inner ear, especially during the embryonic period, is essential. The inner ear was once believed to be “immune-privileged” given that IgG concentrations in the perilymph was as low as that in the cerebrospinal fluid and no lymphatic drainage or lymphoid tissue was present inside the inner ear (3, 4). However, recent studies have revealed the presence of immune-competent cells in the cochlea, which are referred to as resident macrophages in the cochlea (5, 6). Tissue resident macrophages are distributed in virtually all tissues throughout the body and play a central role in both tissue homeostasis and inflammation, completing tissue-specific functions, and protecting the organs and tissue from infection (7, 8).

Regarding ontogeny of tissue resident macrophages, researchers have debated for decades whether resident macrophages were continuously and predominantly repopulated by blood-circulating monocytes, which arise from progenitors in the adult bone marrow (BM) (8). However, several studies have recently revealed that resident macrophages in the steady state have heterogeneous origin among tissues. The homeostatic contribution of circulating monocytes to macrophage populations seems to be restricted to a few specific tissues, including the gut, dermis, and heart, with a turnover rate unique to each tissue in the steady state (8–11). Alternatively, many resident macrophage populations arise from embryonic precursors that reside in these tissues prior to birth and maintain themselves locally throughout adulthood, independent of a major contribution from BM-derived precursors (8). In the steady state, resident macrophages in adult tissues have three major origins, including the yolk sac macrophage, fetal liver monocytes, and BM monocytes (8). As for the functional differences among macrophages derived from the three different origins, it is suggested that there might be some difference in gene expression of macrophages depending on their origins according to the study comparing the gene expression profiles in repopulated bone marrow-derived macrophages after genotoxic irradiation (12) or conditional depletion of macrophages (13). It is also reported that the capacity for self-maintenance (8) or the involvement to pancreatic tumor growth (14) is dominant in macrophages of embryonic origin, whereas the capacity to produce TNF during DSS-induced colitis (15) or Toxoplasma infection (16) is limited to macrophages derived from BM monocyte. However, difference in the role of macrophages of each origin are yet to be elucidated. The proportion of resident macrophages according to each origin differs depending on developmental stages and tissues. For example, most of the microglia in the brain come from the yolk sac macrophage, whereas macrophages from the other two origins contribute little in any stage of life (17). In contrast, although resident macrophages in the gut are derived from the yolk sac during the early embryonic stage, monocytes derived from the fetal liver subsequently comprise most of the resident macrophages in the gut at birth, with most of the resident macrophages ultimately being supplied by the BM during adulthood (8, 10). Regarding resident macrophages in the cochlea, previous reports have shown that at least part of the macrophages in the cochlea are recruited from BM precursors in the steady state (6, 18, 19), in local surgical stress (6), and after noise exposure (18) in adult mice. However, no studies have report the origins of embryonic cochlear macrophages.

The present study examined the development and distribution of resident macrophages in the developing mouse cochlea to elucidate the early spatial and temporal development of cochlear resident macrophages. Colony stimulating factor-1 (Csf1) signaling regulates the survival, proliferation, and differentiation of resident macrophages (20), while its receptor (Csf1 receptor, Csf1r) has been reported to be indispensable for macrophage development from fetal monocytes (17, 21). We therefore took advantage of *Csf1r-null* mice, focusing particularly on the origin and settlement of resident macrophages in the early stages of cochlear development.

## Materials and Methods

### Animals

Pregnant female ICR mice at gestational day 9–19 and pups at postnatal day (P) 1–21 were purchased from Japan SLC, Inc. (Hamamatsu, Japan). *Csf1r*-null mice (22) were kindly provided by Dr. Issay Kitabayashi, National Cancer Center Japan, Tokyo, Japan. Genotyping for *Csf1r*-null allele was performed as previously reported (18). Transgenic mice carrying the Sox10-IRES-Venus allele were also bred as previously reported (23). All animals were maintained under conventional conditions at the Institute of Laboratory Animals, Kyoto University Graduate School of Medicine. All experimental protocols were approved by the Animal Research Committee, Kyoto University Graduate School of Medicine and conducted in accordance with the National Institutes of Health Guide for the Care and Use of Laboratory Animals.

### Preparation of Frozen Sections

#### Fetal Mice

Under general anesthesia using medetomidine, midazolam, and medetomidine butorphanol, fetal mice from E9.5 to E17.5 were extracted from the uterus and immediately decapitated. Whole heads were immersed in 4% paraformaldehyde in phosphate buffer overnight at 4°C and then cryoprotected with 30% sucrose in phosphate buffered saline (PBS) overnight. Specimens were prepared as cryostat sections (10 μm in thickness). Midmodiolar sections were provided for histological analyses.

#### Neonatal Mice

Neonatal mice from P0 to P6 were decapitated soon after euthanization, and whole heads were immersed in 4% paraformaldehyde in phosphate buffer overnight at 4°C and then cryoprotected with 30% sucrose in PBS overnight. Specimens were prepared as cryostat sections (10 μm in thickness). Midmodiolar sections were provided for histological analyses.

#### Mice at P21

Under general anesthesia using medetomidine, midazolam, and medetomidine butorphanol, mice were perfused intracardially with ice-cooled phosphate-buffered saline (PBS), followed by 4% paraformaldehyde in phosphate buffer. Temporal bones were collected and immersed in the same fixative for 4 h at 4°C. Samples were decalcified with 10% ethylenediaminetetraacetic acid in phosphate buffer and cryoprotected with 30% sucrose in PBS. Specimens were prepared as cryostat sections (10 μm in thickness). Midmodiolar sections were provided for histological analyses.

### Immunohistochemistry

Cryostat sections were immersed in blocking solution containing 10% goat serum for 30 min and incubated with a primary antibody at 4°C overnight. Macrophages were labeled according to antibodies for ionized calcium-binding adapter molecule 1 (Iba1), which is specific for microglia/macrophages (24), CD11b, which is a monocyte/macrophage-specific glycoprotein, F4/80, which is a marker for resident macrophages (25), and macrosialin CD68, which is highly expressed in macrophages and other mononuclear phagocytes (26). Ki67 and Phospho-histone H3 (PHH3) were used as cell proliferation markers. The primary antibodies used herein included rabbit anti-Iba1 (1:1,000; Wako Pure Chemicals, Osaka, Japan), rat anti-F4/80 (1:2,000; A3-1; Bio-Rad Laboratories, Inc., Hercules, CA), rat anti-CD68 (1:1,000; FA-11; Serotec, Oxford, United Kingdom), rat anti-CD11b (1:500; M1/70; BD Biosciences, San Jose, CA, USA), rabbit anti-Ki67 (1:500; SP6; Thermo Fisher Scientific K.K., Tokyo, Japan), and mouse anti-pHH3 (1:200; 6G3; Cell Signaling Technology Japan, K.K., Tokyo, Japan) antibodies. Localization of primary antibodies was visualized using secondary antibodies conjugated with Alexa Fluor 488, 546, 633, or 647 (1:500; Molecular Probes, Invitrogen, Carlsbad, CA, USA). Cell nuclei and actin filaments were counterstained with 4′,6-diamidino-2-phenylindole dihydrochloride (DAPI; Invitrogen) and Alexa 633-labeled phalloidin (Invitrogen), respectively. Negative controls lacked primary antibody labeling. Fluorescent images were acquired using a Leica TCS SPE (Leica Microsystems, Wetzlar, Germany).

### Data Analysis of Histological Samples

#### Quantification of Macrophage Subtypes and Proliferating Cells

After quantifying Iba1-, CD11b-, pHH3-, and Ki67-positive cells, each cell type was defined as follows. An Iba1-positive cell had both a nucleus and cell body that were positive for DAPI and Iba1 immunohistostaining, respectively. A CD11b-positive cell had both a nucleus and cell membrane that were positive for DAPI and CD11b immunohistostaining, respectively. A pHH3- or Ki67-positive cell had a nucleus that was positive for both DAPI and pHH3 or Ki67, respectively.

The proportion of pHH3 -positive cells in Iba1-positive cells and the proportion of Ki67-positive cells in F4/80-positive cells were defined as the number of cells double positive for pHH3 and Iba1 staining divided by the number of Iba1-positive cells and as the number of cells double positive for Ki67 and F4/80 staining divided by the number of F4/80-positive cells, respectively.

#### Cell Density of Iba1- or CD11b-Positive Cells

Cell density of Iba1- or CD11b-positive cells was defined as the number of Iba1- or CD11b-positive cells per square millimeter of the specimen. The whole area of the cochlea was defined as the inside area of the bony cochlea. The area of the bony cochlea, spiral ganglion, spiral ligament, and stria vascularis was determined through phalloidin and DAPI staining.

Cell density data of Iba1- or CD11b-positive cells from one mouse consisted of at least five inconsecutive specimen sections. Cell density in the spiral ganglion, spiral ligament, and stria vascularis was defined as the number of cells per 1 mm-square of the spiral ganglion, spiral ligament, or stria vascularis, whereas that in the cochlear mesenchyme and on the intraluminal surface of the perilymphatic space was defined as the number of cells per 1 mm-square of bony cochlea.

### Statistical Analysis

Statistical analysis was performed using GraphPad Prism software (Prism 8 for Windows; GraphPad Software Inc., San Diego, CA). One-way analysis of variance (ANOVA) with Tukey's multiple comparisons test, Sidak's multiple comparisons test, or unpaired *t*-tests was used for parametric analyses. Results are presented as mean values ± standard errors. A two-tailed *p* < 0.05 was considered statistically significant.

## Results

### Tissue Macrophage Emergence in the Mesenchyme Around the Developing Otocyst Between E9.5 and E10.5

**Figure 1:**
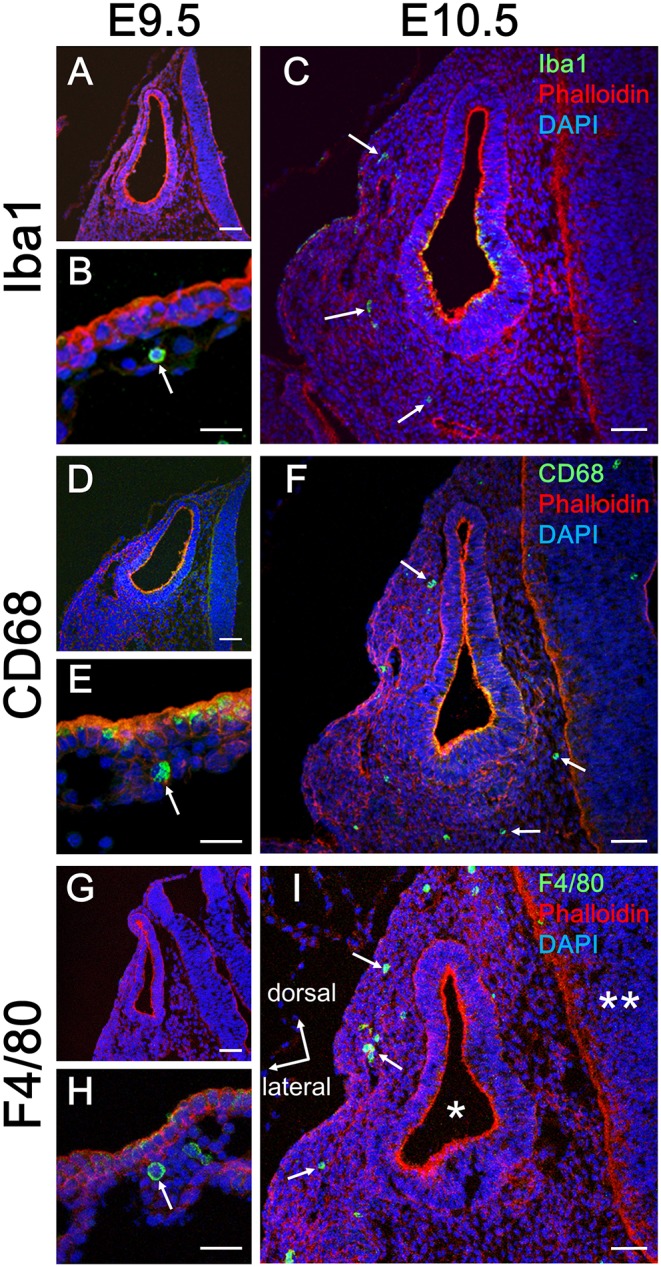
Emergence of macrophages around the otocyst at E10.5. At E9.5, although no macrophages were observed around the otocyst, which were labeled according to macrophage marker, Iba1, CD68, or F4/80 **(A,D,G)**, they were observed in the yolk sac at the same period **(B,E,H)**. At E10.5, however, macrophages resided around the otocyst, which were labeled according to macrophage marker, Iba1, CD68, or F4/80 **(C,F,I**, arrows). Asterisk = otocyst; double asterisk = neural tube. Scale bars = 50 μm in **(A,C,D,F,G,I)** and 20 μm in **(B,E,H)**.

Initially, emergence distribution of macrophages in the embryonic mouse cochlea was examined using immunohistochemistry for Iba1, CD68, and F4/80. In embryonic mice, resident macrophages appear at E10.5 in various tissues, including the brain (17) and skin (27). At E9.5, neither Iba1-, CD68-, nor F4/80-positive macrophages were observed around the otocyst (Figures 1A,D,G), whereas macrophage precursors labeled with Iba1, CD68, or F4/80 were observed in the yolk sac at E9.5 (Figures 1B,E,H). At E10.5, however, macrophages were observed around the otocyst labeled with either anti-Iba1, CD68, or F4/80 antibodies (Figures 1C,F,I). The aforementioned data indicate that resident macrophages migrate and settle in the mesenchyme surrounding the otocyst as early as E10.5. Moreover, hematopoiesis at E9.5 or E10.5 is thought to be mainly provided by yolk sac. Therefore, these results suggest that progenitors of cochlear resident macrophages could be supplied by the yolk sac given that primitive macrophages first appear in the blood islands of the mouse yolk sac at E9 and hematopoiesis in the fetal liver or aorta-gonad-mesonephron area starts from E10.5 onward (28).

### Dynamic Changes in the Distribution of Resident Macrophages in the Developing Cochlea

**Figure 2:**
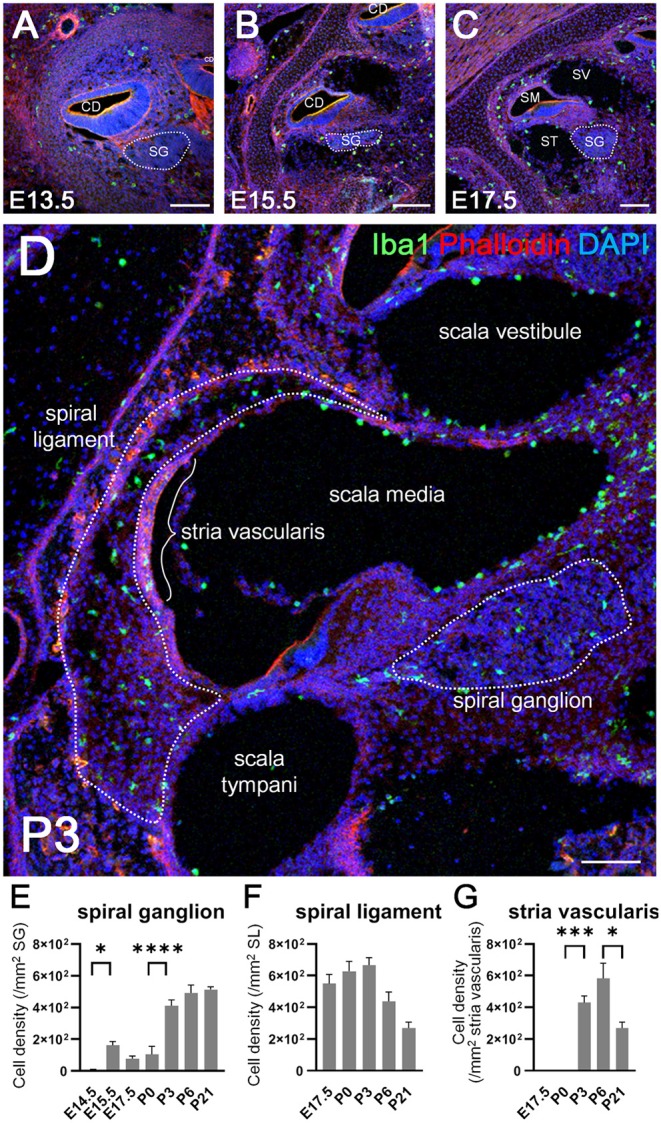
Distribution and cell density of Iba1-positive macrophages in the cochlea at embryonic, neonatal, and adult stages. **(A–D)** Immunohistochemistry findings of the cochlea at E13.5 **(A)**, E15.5 **(B)**, E17.5 **(C)**, and P3 **(D)** using anti-Iba1 antibody. During the embryonic stages, Iba1-positive macrophages were distributed in the cochlear mesenchyme **(A,B)**. At E17.5 or P3, Iba1-positive macrophages were distributed widely in the cochlea, especially in the spiral ligament, spiral ganglion, and stria vascularis **(C,D)**. **(E–G)** Iba1-positive cell density in the spiral ganglion, spiral ligament, and stria vascularis at various developmental stages. In the spiral ganglion, Iba1-positive cell density increased as developmental stages progressed, whereas in the spiral ligament, the same achieved a peak at P3 and subsequently decreased during the neonatal stages (from P3 onward). On the other hand, Iba1-positive cell in the stria vascularis were observed only after birth and its peak was observed at neonatal stages. CD, cochlear duct; SG, spiral ganglion; SM, scala media; ST, scala tympani; SV, scala vestibuli. Scale bars = 100 μm. Statistical differences were assessed using one-way ANOVA with Sidak's multiple comparisons test. ^*^p < 0.05; ^***^p = 0.001; ^****^p < 0.0001.

We next examined the distribution of Iba1-positive macrophages in the developing cochlea following their emergence around the otocyst at E10.5. In the developing embryonic cochlea from the period E13.5 to E15.5, Iba1-positive macrophages were observed in the cochlear mesenchyme (Figures 2A,B). During the perinatal, neonatal, and adult stages, Iba1-positive macrophages were widely distributed in the cochlea, particularly in the spiral ganglion, spiral ligament, and stria vascularis, as well as on the intraluminal surface of perilymphatic space or the mesenchyme (Figures 2C,D). Cell density of Iba1-positive macrophages varied depending on the region and embryonic stages (Figures 2E–G). It was 5.29 ± 5.29 at E14.5, 162 ± 23.6 at E15.5, 77.5 ± 16.7 at E17.5, 105 ± 50.8 at P0, 411 ± 36.6 at P3, 492 ± 49.9 at P6, and 513 ± 17.8 at P21 in the spiral ganglion (/mm^2^, mean ± SEM), 550 ± 57.2 at E17.5, 628 ± 61.9 at P0, 666 ± 47.3 at P3, 438 ± 58.8 at P6, and 270 ± 37.3 at P21 in the spiral ligament (/mm^2^, mean ± SEM), and 0 ± 0 at E17.5, 0 ± 0 at P0, 430 ± 41.5 at P3, 583 ± 96 at P6, and 356 ± 32 in the stria vascularis (/mm^2^, mean ± SEM). The density of Iba1-positive cells in the spiral ganglion increased as developmental stages progressed, whereas the density of Iba1-positive macrophages in the spiral ligament achieved a peak at P3 and subsequently decreased during the neonatal stages (from P3 onward). On the other hand, Iba1-positive macrophages in the stria vascularis were observed only after birth and its peak was observed at neonatal stages. Statistical analyses demonstrated significant differences in Iba1-positive macrophage density among the developmental stages in each part of the cochlea, such as the spiral ganglion, spiral ligament, and stria vascularis (one-way ANOVA, *p* < 0.0001, *p* = 0.0028, and *p* < 0.0001, respectively). *Post-hoc* analysis using Sidak's multiple comparisons test between two sequential developmental stages showed significant changes in Iba1-positive macrophage density between E14.5 and E15.5 and between P0 and P3 in the spiral ganglion (*p* = 0.0128 and *p* < 0.0001, respectively) and between P0 and P3 and between P6 and P21 in the stria vascularis (*p* = 0.0001 and 0.0378, respectively). Taken together, the aforementioned data indicated dynamic changes in the distribution of cochlear resident macrophages depending on cochlear tissue area or developmental stages.

### *In situ* Proliferation Capacity of Cochlear Resident Macrophages

**Figure 3:**
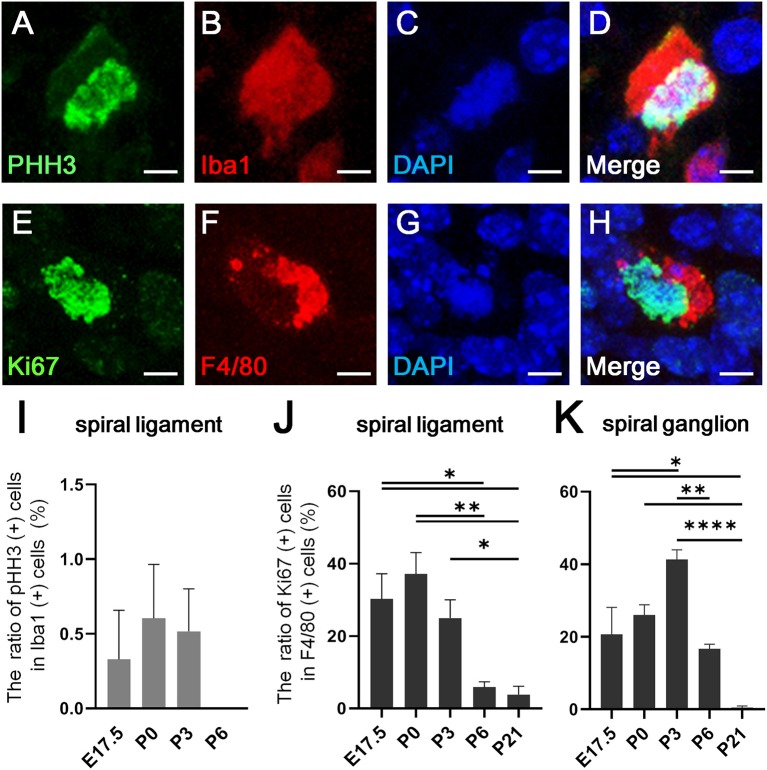
*In situ* proliferative capacity of resident macrophages in the cochlea. **(A–D)** A typical cell double-positive for pHH3 and Iba1, which was found in the cochlear mesenchyme at E13.5, is shown by immunostaining for pHH3 **(A)**, Iba1 **(B)**, DAPI **(C)**, and Merge **(D)**. **(E–H)** A typical cell double-positive for Ki67 and F4/80, which was also found in the cochlear mesenchyme at E13.5, is shown by immunostaining for Ki67 **(E)**, F4/80 **(F)**, DAPI **(G)**, and Merge **(H)**. **(I)** The proportion of pHH3 and Iba1 double-positive cells among Iba1-positive cells in the spiral ligament. **(J,K)** The proportion of Ki67 and F4/80 double-positive cells among F4/80-positive cells in the spiral ligament **(J)** and spiral ganglion **(K)**. SL, spiral ligament; SG; spiral ganglion. Scale bars = 10 μm in **(A–H)**. Statistical differences were assessed using one-way ANOVA with Tukey's multiple comparisons test. ^*^p < 0.05; ^**^p < 0.01; ^****^p < 0.0001.

Recent studies have shown that resident macrophages proliferate *in situ* and self-maintain locally (29, 30) in organs, such as the brain (31), lungs (29), and heart (7). However, studies have yet to determine whether cochlear resident macrophages proliferate *in situ*. Thus, we assessed the *in situ* proliferation capacity of cochlear resident macrophages. Accordingly, pHH3- and Ki67-positive macrophages were found in the developing cochlea at the embryonic and neonatal stages (Figures 3A–H). The percentage of pHH3-positive cells among Iba1-positive cells was <1% in the spiral ligament (Figure 3I), while no pHH3-positive cells among Iba1-positive cells were found in the spiral ganglion at any developmental stage (from E14.5 to P21). The percentage of pHH3-positive cells among Iba1-positive cells was 0.329 ± 0.329 at E17.5, 0.604 ± 0.361 at P0, 0.516 ± 0.284 at P3, and 0.00 ± 0.00 at P6 in the spiral ligament (%, mean ± SEM). The percentage of Ki67-positive cells among F4/80-positive cells achieved a peak at P0 in the spiral ganglion and at P3 in the spiral ligament (Figures 3J,K). It was 30.2 ± 6.99 at E17.5, 37.1 ± 5.92 at P0, 25.0 ± 5.01 at P3, 5.85 ± 1.52 at P6, and 3.84 ± 2.29 at P21 in the spiral ligament (%, mean ± SEM), and 20.7 ± 7.44 at E17.5, 26.0 ± 2.84 at P0, 41.3 ± 2.67 at P3, 16.6 ± 1.37 at P6, and 0.472 ± 0.472 at P21 in the spiral ganglion (%, mean ± SEM). Statistical analyses showed no significant difference in the proportion of pHH3-positive cells among Iba1-positive macrophages across different developmental stages in the spiral ligament (one-way ANOVA, *p* = 0.467). In contrast, significant differences were observed in the proportion of Ki67-positive cells among F4/80-positive cells across developmental stages in both the spiral ligament and spiral ganglion (one-way ANOVA, *p* = 0.0005 and *p* < 0.0001, respectively). In the spiral ligament, significant differences were demonstrated in the proportion of Ki67-positive macrophages between E17.5 and P6 or P21 (Tukey's multiple comparisons test, *p* = 0.0199 or 0.0113, respectively), between P0 and P6 or P21 (*p* = 0.0028 or 0.0016, respectively) and between P3 and P21 (*p* = 0.049). In the spiral ganglion, significant differences in the proportion of Ki67-positive macrophages were observed between E17.5 and P3 or P21 (Tukey's multiple comparisons test, *p* = 0.0124 or 0.0142, respectively), between P0 and P21 (*p* = 0.0021), between P3 and P6 (*p* = 0.0029), and between P3 and P21 (*p* < 0.0001). The aforementioned data suggest that resident macrophages in the spiral ligament or spiral ganglion of developing cochlea have capacity for *in situ* proliferation and self-renewal.

### Distinction Between Macrophages in the Embryonic Cochlea and Cell Population of Neural Crest Origin

**Figure 4:**
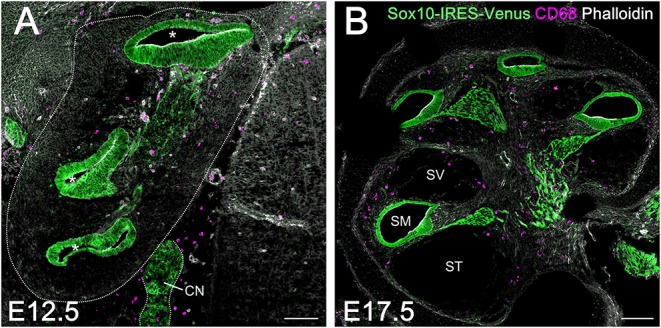
Comparison between resident macrophages and cell population of neural crest origin in the embryonic cochlea using Sox10-IRES-Venus mice. Macrophages expressing CD68 in the embryonic cochlea were distinct from Sox10-Venus positive cells at E12.5 **(A)** and E17.5 **(B)**, indicating that resident macrophages in the embryonic cochlea did not originate from the neural crest. A dotted line shows the lateral edge of the cochlea. Asterisks, cochlear duct; CN, cochlear nerve; SM, scala media; ST, scala tympani; SV, scala vestibuli. Scale bars = 100 μm.

A large population of perivascular cells has been reported to be present in the area around the blood-labyrinth barrier in the stria vascularis of the lateral cochlear wall in adult mice. The cells are identified as perivascular resident macrophages or perivascular-resident macrophage-like melanocytes given their positivity for several macrophage markers, including F4/80, CD68, and CD11b (19, 32). Considering that melanocytes are generally thought to originate from the neural crest, we examined whether macrophages that reside in the spiral ligament or spiral ganglion of the embryonic cochlea were derived from the neural crest. We took advantage of transgenic mice carrying the Sox10-IRES-Venus cassette to visualize the cell population of neural crest origin and compare it to CD68-positive macrophages. At E12.5 and E17.5, macrophages expressing CD68 in the embryonic cochlea were distinct from Sox10-Venus positive cells (Figures 4A,B). Although Sox10 not only marks neural crest-derived cells but also delaminating neuroblasts and otic placode-derived prosensory cells (23, 33), such data indicate that resident macrophages in the embryonic cochlea do not originate from the neural crest, while presumably cochlear resident macrophages expressing macrophage markers, including F4/80, CD68, or Iba1, observed in the spiral ligament and spiral ganglion were distinct from perivascular resident macrophages or perivascular-resident macrophage-like melanocytes.

### Fetal Liver Hematopoiesis as an Alternative Source of Cochlear Macrophage Precursors

**Figure 5:**
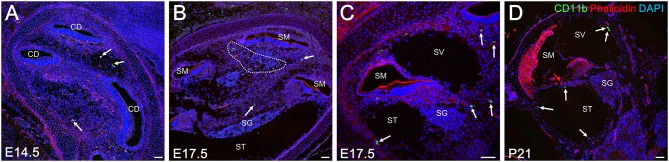
Distribution of CD11b-positive cells in the cochlea at embryonic, neonatal, and adult stages. From E14.5 onward, CD11b-positive cells were observed in the cochlear mesenchyme (**A**, arrows). At E 17.5, CD11b-positive cells were observed in the mesenchyme around the cochlear modiolus (**B**, arrows) or on the intraluminal surface of the scala tympani and scala vestibuli (**C**, arrows). At P21, CD11b-positive cells were found mainly on the intraluminal surface of the scala tympani and scala vestibuli (**D**, arrows). CD, cochlear duct; SG, spiral ganglion; SM, scala media; ST, scala tympani; SV, scala vestibuli. Scale bars = 50 μm.

As mentioned previously, embryonic hematopoiesis is first established in yolk sac at E9 followed by migration of hematopoietic progenitors into the fetal liver from E10.5 onward. Therefore, the distribution of CD11b-positive cells in the developing cochlea were subsequently examined to determine whether fetal liver hematopoiesis could serve as a source of resident macrophage precursors (34). Unlike Iba1-, CD68-, or F4/80-positive cells, CD11b-positive cells were rarely found around the otocyst or the cochlea by E13.5. Moreover, a few CD11b-positive cells were observed in the cochlear mesenchyme from E14.5 onward (Figure 5A). At E17.5, the embryonic stage at which the perilymphatic space was clearly formed, CD11b- positive cells were found in the cochlear mesenchyme around the cochlear modiolus, especially in the apical turn, or on the intraluminal surface of the scala tympani and scala vestibuli (Figures 5B,C). At P21, CD11b-positive cells were mainly observed on the intraluminal surface of the scala tympani and scala vestibuli (Figure 5D). The distribution of CD11b-positive cells demonstrated a unique pattern distinct from that of Iba1-, CD68-, or F4/80-positive cells. Thus, the aforementioned data suggest that fetal liver homeostasis could serve as an alternative source of cochlear macrophage precursors.

### Transition of Cochlear Macrophage Phenotype From Fetal Liver-Derived Circulating Monocyte Precursors to Tissue Resident Macrophages

**Figure 6:**
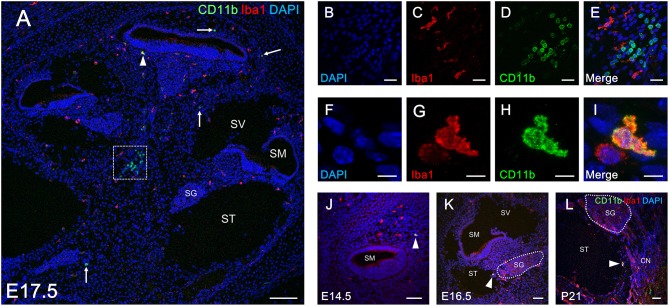
Double immunostaining for Iba1 and CD11b in the cochlea at perinatal and adult stages. Distribution of Iba1-postive macrophages and CD11b-positive cells (**A**, arrows) throughout the entire cochlea at E17.5 **(A)**. Typical cells single-positive for Iba1 and CD11b in an inset of **(A)** are shown by immunostaining for DAPI **(B)**, Iba1 **(C)**, CD11b **(D)**, and Merge **(E)**. A portion of Iba1- or CD11b-positive cells in the cochlea at E17.5 were double-positive for both markers (**A**, arrowhead). A typical cell double positive for Iba1 and CD11b is shown by immunostaining for DAPI **(F)**, Iba1 **(G)**, CD11b **(H)**, and Merge **(I)**. Iba1 and CD11b double-positive cells in the cochlea were found at all developmental stages (from E14.5 to P21) (**J–L**, arrowheads). These findings suggest a direct transition in macrophage phenotype from CD11b-positive macrophage precursors to Iba1-positive macrophages. CN, cochlear nerve, SG, spiral ganglion; SM, scala media; ST, scala tympani; SV, scala vestibuli. Scale bars = 100 μm in **(A)**, 20 μm in **(B–E)**, 10 μm in **(F–I)**, and 50 μm in **(J–L)**.

The findings regarding the distribution of CD11b-positive cells in the developing cochlea led us to hypothesize that CD11b-positive precursors derived from fetal liver also settle and differentiate as tissue resident macrophages in the cochlea. To assess distribution differences between cochlear resident macrophages and precursors derived from the fetal liver, we next performed double immunostaining for Iba1 and CD11b using developing cochleae. As expected from the results presented in Figures 2, 5, the spatial distribution patterns of Iba1- and CD11b-positive cells were totally distinct from each other (Figure 6A). However, some cochlear macrophages expressed both Iba1 and CD11b (Figures 6A,F–I). The aforementioned data suggest that at least three types of monocytes/macrophage linage cells are present in the cochlea: Iba1 or CD11b single-positive cells (Figures 6A–E), Iba1, and CD11b double-positive cells (Figures 6A,F–I). Our data also imply the possibility for direct transition of macrophage phenotype from CD11b-positive macrophage precursors to Iba1-positive macrophages. Moreover, Iba1, and CD11b double-positive cells were exclusively observed in the mesenchyme of the cochlear modiolus or on the intraluminal surface of perilymphatic space at any developmental stage between E14.5 and P21 (Figures 6A,J–L).

### Essential Role of Csf1 Signaling in Cochlear Resident Macrophage Development and the Independent Contribution of Fetal Liver Hematopoiesis to Monocyte Precursors in the Cochlear Modiolus

**Figure 7:**
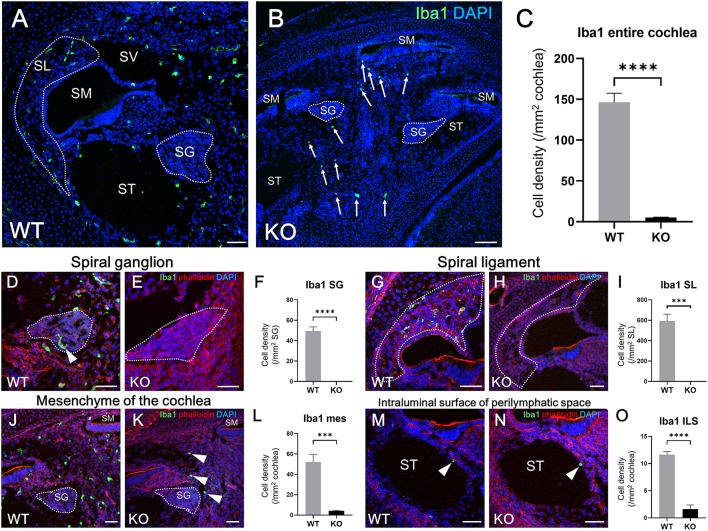
Distribution and cell density of Iba1-positive cells in the cochlea of *Csf1r-null* mice at E17.5. Typical immunohistochemistry findings using anti-Iba1 antibody in the entire cochlea **(A,B)**, spiral ganglion **(D,E)**, spiral ligament **(G,H)**, mesenchyme **(J,K)**, and intraluminal surface of the perilymphatic space **(M,N)** in wildtype and *Csf1r*-null mice. The cell density of Iba1-positive macrophages in the entire cochlea **(C)**, spiral ganglion **(F)**, spiral ligament **(I)**, cochlear mesenchyme **(L)**, and intraluminal surface of the perilymphatic space **(O)** in both wildtype and *Csf1r*-null mice. *Csf1r*-null mice had a significantly lower Iba1-positive cell density than wildtype mice in any region of the cochlea, suggesting that Csf1 signaling plays essential roles in the development of cochlear resident macrophages originating from the yolk sac. WT, wildtype mice; KO, *Csf1r*-null mice; SG, spiral ganglion; SL, spiral ligament; SM, scala media; ST, scala tympani; SV, scala vestibuli; mes, mesenchyme; ILS, intraluminal surface of perilymphatic space. Scale bars = 50 μm. Statistical differences were assessed using unpaired *t*-tests. ^***^p = 0.001; ^****^p < 0.0001.

**Figure 8:**
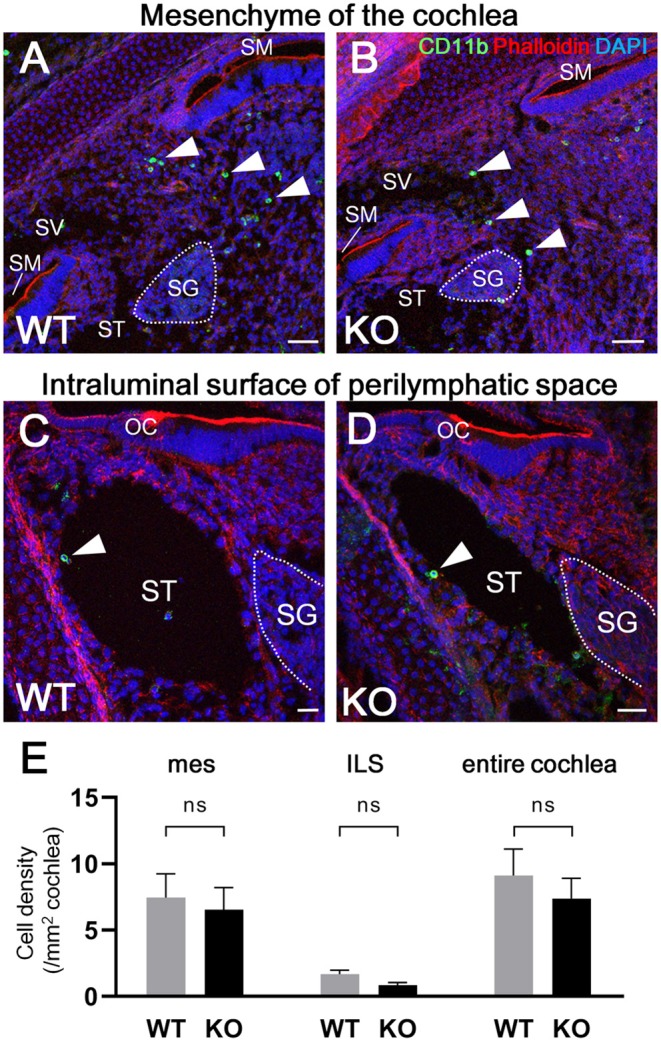
Distribution of CD11b-positive cells in the cochlea of *Csf1r*-null mice. Typical immunohistochemistry findings using anti-CD11b antibody in the cochlear mesenchyme **(A,B)** and perilymphatic space **(C,D)** in wildtype and *Csf1r*-null mice. No significant differences in CD11b-positive cell density were observed between wildtype and *Csf1r*-null mice in any region, including the mesenchyme of the cochlear modiolus, intraluminal surface of the perilymphatic space, and entire cochlea **(E)**, suggesting that fetal liver hematopoiesis contributes to monocyte precursors in the cochlear modiolus independent of Csf1 signaling. mes, mesenchyme; ILS, intraluminal surface of perilymphatic space. Scale bars = 50 μm in **(A,B)** and 20 μm in **(C,D)**. Statistical differences were assessed using unpaired *t*-tests. WT, wildtype mice; KO, *Csf1r*-null mice.

According to the results of previous studies, the development of macrophages originating from the yolk sac largely depend on Csf1 signaling, whereas the development of fetal liver monocytes is independent of Csf1 signaling (17, 21, 34). To study the role of Csf1 signaling or yolk sac hematopoiesis in the development of cochlear resident macrophages and their precursors, we took advantage of *Csf1r*-null mice (22) and assessed the distribution of Iba1- and CD11b-positive cells in the cochlea at E17.5. While Iba1-positive macrophages were widely distributed over the entire cochlea of wildtype mice (Figure 7A), fewer Iba1-positive macrophages were observed in the mesenchyme of cochlear modiolus or the intraluminal surface of perilymphatic space in *Csf1r*-null mice (Figure 7B,J,K,M,N). Particularly in the spiral ganglion and spiral ligament, no Iba1-positive macrophages were found in the cochlea of *Csf1r*-null mice (Figures 7D,E,G,H). Cell density of Iba1-positive macrophages at E17.5, in wildtype and *Csf1r*-null mice, respectively, was 146 ± 11.3 and 5.13 ± 0.516 in the entire cochlea (/mm2, mean ± SEM), 49.3 ± 4.17 and 0.00 ± 0.00 in the spiral ganglion (/mm2, mean ± SEM), 594 ± 65.6 and 0.00 ± 0.00 in the spiral ligament (/mm2, mean ± SEM), 52.3 ± 7.26 and 4.20 ± 0.428 in mesenchyme cochlear modiolus (/mm2, mean ± SEM), and 11.6 ± 0.559 and 1.60 ± 0.769 on the intraluminal surface of perilymphatic space (/mm2, mean ± SEM). Statistical analysis showed that *Csf1r*-null mice had significantly lower Iba1-positive cell density than wildtype mice in the entire cochlea (unpaired *t*-test, *p* < 0.0001) (Figure 7C) and in any region, including the spiral ganglion, spiral ligament, mesenchyme of cochlear modiolus, and intraluminal surface of perilymphatic space (*p* < 0.0001, *p* = 0.0001, *p* = 0.0006, and *p* < 0.0001, respectively) (Figures 7F,I,L,O). In contrast, the distribution of cochlear CD11b-positive cells in *Csf1r*-null mice demonstrated a spatial pattern identical to that in wildtype mice. In both wildtype and *Csf1r*-null mice, CD11b-positive cells were observed on the intraluminal surface of the perilymphatic space or in the mesenchyme of the cochlear modiolus (Figures 8A–D). Cell density of CD11b-positive cells at E17.5, in wildtype and *Csf1r*-null mice, respectively, was 7.44 ± 1.81 and 6.54 ± 1.68 in the mesenchyme of cochlear modiolus (/mm2, mean ± SEM), 1.68 ± 0.300 and 0.845 ± 0.201 on the intraluminal surface of the perilymphatic space (/mm2, mean ± SEM), and 9.12 ± 1.98 and 7.38 ± 1.53 in the entire cochlea (/mm2, mean ± SEM). Statistical analysis showed that no significant difference in the density of CD11b-positive cells between wildtype and *Csf1r*-null mice in the mesenchyme of the cochlear modiolus, intraluminal surface of the perilymphatic space, and entire cochlea (unpaired *t*-test, *p* = 0.731, 0.066, and 0.5281, respectively) (Figure 8E). Taken together, Csf1 signaling plays an essential role in the development of cochlear resident macrophages originating from the yolk sac. Meanwhile, fetal liver hematopoiesis seems to contribute to monocyte precursors in the cochlear modiolus independent of Csf1 signaling.

## Discussion

The present study revealed that resident macrophages in mice emerge around the otocyst at as early as E10.5. Resident macrophages were distributed in the cochlear mesenchyme during the embryonic stages and subsequently located particularly in the spiral ligament, spiral ganglion, and stria vascularis in the postnatal cochlea. The density of the cochlear macrophages increased as mice grew, peaked around the neonatal stages, and decreased from P3 onward. In addition, cochlear macrophages have *in situ* proliferative capacity especially during the perinatal period. We also identified CD11b-positive monocytes that seemed to be derived from the fetal liver as an alternative cell source of macrophage precursors and are distributed in the mesenchyme of cochlear modiolus or on the intraluminal surface of the perilymphatic space. Although the use of an adequate fate mapping model should be desirable to ascribe a hematopoietic lineage to a type of macrophage, our findings revealed the heterogeneity of resident macrophages in the embryonic cochlea while also suggesting the heterogeneity of their hematopoietic origins.

### Subtypes and Origins of Cochlear Resident Macrophages

**Figure 9:**
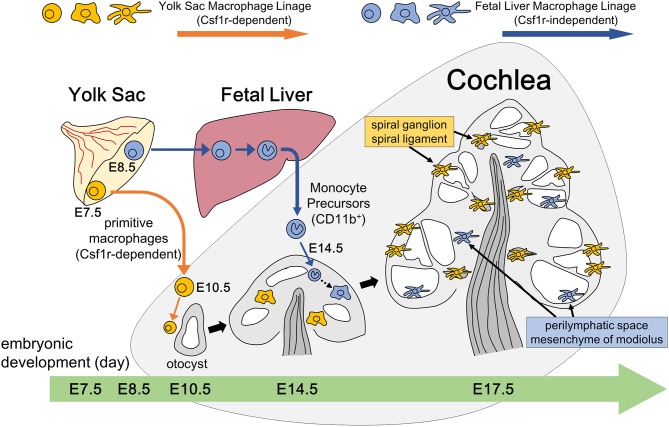
Schematic representation of resident macrophage development in the embryonic cochlea through two different pathways. Two subtypes of resident macrophages are present in the embryonic cochlea: one being Csf1r-dependent macrophages that originate from the yolk sac and the other being Csf1r-independent macrophages that migrate from the fetal liver via systemic circulation. A large portion of the cochlear resident macrophage population is derived from the yolk sac given that macrophages expressing Iba1, CD68, or F4/80 reside in the mesenchyme surrounding the otocyst at as early as E10.5. These macrophages are distributed in the spiral ganglion and spiral ligament at E17.5, which is promoted by the Csf1 signaling. On the other hand, Csf1r-independent macrophages expressing CD11b migrate at as early as E14.5 and reside only in certain parts of the cochlea, such as the mesenchyme of the cochlear modiolus or the intraluminal surface of the perilymphatic space at E17.5.

The present study depicted the spatial and temporal distribution pattern of resident macrophages in the developing cochlea from E9 through postnatal stages. Regarding the emergence of cochlear macrophages, macrophage lineage cells expressing CX3CR1 have been observed around the otocyst at E10.5 in mice (35), which is consistent with the results presented herein. The ontogeny of resident fetal macrophages is believed to be categorized into two pathways (8). Firstly, macrophage progenitors arise from the blood island of the yolk sac at E7.5 and migrate through blood vessels directly to virtually all organs and tissues throughout the entire body at E10.5. Macrophages in other tissues, such as the brain (36) or developing skin (27), have also been observed as early as E10.5. Reports have shown that the development of primitive macrophages derived from the yolk sac depends on Csf1 signaling (17, 21). Secondly, in contrast to the yolk sac-derived primitive macrophages, monocyte progenitors arise from the yolk sac at E8.5 or from the aorta, gonads, and mesonephron regions at E10.5, migrate, and subsequently differentiate into fetal monocytes inside the fetal liver. These monocyte progenitors finally migrate to each tissue at E14.5 and differentiate into mature resident macrophages (8). The findings obtained herein suggest that a large portion of the cochlear resident macrophage population is derived from the yolk sac given that macrophages expressing Iba1, CD68, or F4/80 reside in the mesenchyme surrounding the otocyst at as early as E10.5. Moreover, cochlear analyses in *Csf1r*-null mice demonstrated that the number of macrophages in the spiral ganglion and spiral ligament were substantially decreased, suggesting that the supply of resident macrophages to such areas was dependent on the Csf1 signaling. These results also suggest that resident macrophages in the spiral ganglion and spiral ligament were derived from the yolk sac given that development of macrophages from the yolk sac were mainly promoted by the Csf1 signaling. Although Csf1r is expressed on macrophages derived from the yolk sac and fetal monocyte precursors derived from the fetal liver, migration and generation of macrophage precursors expressing CD11b appeared to be independent of the Csf1 signaling, which is consistent with previous reports (17, 21, 34). Taken together, our findings in *Csf1r*-null mice demonstrate that two subtypes of resident macrophages are present in the embryonic cochlea: one being Csf1r-dependent macrophages that originate from the yolk sac and the other being Csf1r-independent macrophages that migrate from the fetal liver via systemic circulation (Figure 9). Remarkable decrease of Langerhans cell precursors was observed in the skin of Csf1r-deficient mice while the absence of Csf1r did not affect monocyte development in the fetal liver and their recruitment to the skin, and Hoeffel et al. speculate that differentiation from fetal liver monocytes into Langerhans cell precursors is dependent on Csf1r (21). On the other hand, in the present study, we focused on the findings that a few Iba1-positive macrophages were still present in the cochlea of *Csf1r*-null mice and speculated that cochlear macrophages of *Csf1r*-null mice have differentiated from fetal liver monocytes. Csf1r-independent macrophages expressing Iba1 reside only in certain parts of the cochlea, such as the mesenchyme of the cochlear modiolus or the intraluminal surface of the perilymphatic space. Differences between the two types of cochlear macrophages are directly linked to the differences in the cochlear tissue site. In addition, quantitative assessment of resident macrophage density in wildtype and *Csf1r*-null mouse cochleae demonstrated that most of the cochlear macrophages during embryonic stage were Csf1r-dependent macrophages derived from the yolk sac.

### Distribution and *in situ* Proliferative Capacity of Cochlear Resident Macrophages

Our findings on the *in situ* proliferative capacity of Iba1-positive cochlear macrophages suggest that the high density of macrophages in the neonatal cochlea might be due to *in situ* proliferation but not recruitment of macrophages from the systemic circulation. The percentage of pHH3-positive resident macrophages in the peritoneal cavity of mice has been reported to be 0.011% at 12–16 weeks of age to 0.25% at 2 weeks of age (37), with another report suggesting the same to be around 1.5% in the fetal lung (38). In addition, cardiac resident macrophages were reported to lose their self-renewal capacity with age (9). Accordingly, findings regarding the percentage of macrophages throughout the cell cycle presented in the aforementioned reports were consistent with those found herein. Concerning self-renewal of the macrophage lineage, two mechanisms of proliferation have been assumed. A number of resident macrophage-dedicated progenitor cells distinct from mature cells are thought to undergo asymmetrical cell division and subsequent terminal maturation into fully differentiated macrophages. In contrast, several studies have suggested that mature differentiated macrophages may undergo self-renewal through a mechanism whereby a fully differentiated macrophage proliferates and gives rise to equally mature macrophages (29, 39). In addition to self-renewal, Okano et al. reported that cochlear resident macrophages in adult mice are slowly replaced by circulating monocyte precursors supplied through hematopoiesis in the BM (6). Therefore, three major sources supplying resident macrophages in the cochlea seem to be present in adults: the yolk sac, fetal liver, and BM. The proportion of macrophages from the three different origins can change depending on the developmental stage of mice as has been reported in other tissues (8). Guilliams et al. quantitatively showed that alveolar fetal monocytes differentiate into mature alveolar macrophages with decreasing CD11b and increasing F4/80 expression using flow cytometry (40). Similarly, findings on double staining for CD11b and Iba1 in the present study suggested, at least in part, that CD11b single-positive fetal monocytes derived from the fetal liver undergo phenotype conversion into Iba1 single-positive mature macrophages through CD11b and Iba1 double-positive intermediate cells. Moreover, Iba1 and CD11b double-positive cells were observed from E14.5 to P21, which is consistent with results presented in previous reports that show fetal monocytes emerging in the fetal liver at around E12.5 and reaching systemic circulation by E13.5 to colonize every tissue except the brain (21, 34). Furthermore, the fact that CD11b-positive cells in wildtype mice were limited to the mesenchyme of the cochlear modiolus or intraluminal surface of the cochlea supports our hypothesis that macrophages derived from fetal liver monocytes are restricted to such areas.

### Potential for Resident Macrophages in the Embryonic Cochlea to Be a Therapeutic Target

Previous studies have suggested that cochlear macrophages play different roles, such as the regulation of auditory glial cell number during postnatal development *in vivo* (41) and phagocytosis of damaged hair cells *in vitro* (35). However, specific macrophage roles in the embryonic cochlea currently remain unknown. Although the results presented herein do not directly demonstrate the roles of resident macrophages in the developing cochlea, the finding that two subtypes of cochlear resident macrophages reside in different parts of the cochlea may suggest different roles depending on the subtype. Regarding the development of a novel therapeutic treatment for congenital hearing loss, several studies have suggested macrophage-targeted therapies for the treatment of inflammatory diseases. One of the most exploited approaches has been facilitating macrophage phagocytosis of a loaded micro vehicle, which is then passively targeted to the site of inflammation due to mounting immune response (42). Modulation and reprogramming of macrophages is considered as a promising antitumor strategy. Hutter et al. showed that anti-CD47 treatment is efficacious against various brain tumors primarily by inducing tumor phagocytosis of macrophages (43). Macrophage depletion is another option to consider for therapeutic approach in pathologic condition, which based on the use of either depleting antibodies, such as anti-Csf1r, or molecules exerting a specific toxicity against macrophages, such as bisphosphonates and trabectedin (44). In cervical and mammary carcinoma mouse models, the depletion of tumor-associated macrophages, obtained by means of a highly selective Csf1r inhibitor, resulted in the arrest or delay of tumor growth (45). In conclusion, the modulation of the Csf1 signaling, which had a considerable impact on the number and distribution of cochlear macrophages in the present study, might lead to the therapeutic treatment of diseases by controlling the dynamics of cochlear resident macrophages later on.

## Data Availability Statement

The raw data supporting the conclusions of this manuscript will be made available by the authors, without undue reservation, to any qualified researcher.

## Ethics Statement

The animal study was reviewed and approved by the Animal Research Committee, Kyoto University Graduate School of Medicine.

## Author Contributions

TO and KO designed and conceived the experiments. IK, KN, and TO performed the experiments and wrote the manuscript. TM developed mice used in the study. IK and TO analyzed the data. TO obtained funding.

### Conflict of Interest

The authors declare that the research was conducted in the absence of any commercial or financial relationships that could be construed as a potential conflict of interest.
